# Comparative effects of reactive and planned agility training on physical performance, internal load and enjoyment in youth soccer players

**DOI:** 10.1038/s41598-026-41891-8

**Published:** 2026-02-27

**Authors:** Nidhal Doua, Hamza Marzouki, Okba Selmi, Bilel Cherni, Rachad Djeddi, Dan Iulian Alexe, Umut Canli, Michal Wilk, Ezdine Bouhlel, Eduard-Robert Sakizlian

**Affiliations:** 1 High Institute of Sport and Physical Education of Kef, University of Jendouba, Kef, Tunisia; 2 Research Unit: Sport sciences, Health and Movement, University of Jendouba, Kef, Tunisia; 3 Tunisian Research Laboratory “Sports Performance Optimization” (LR09SEP01), National Center of Medicine and Science in Sports (CNMSS), Tunis, Tunisia; 4 Department of Physical and Occupational Therapy, “Vasile Alecsandri” University of Bacau, 600115 Bacău, Romania; 5 Department of Physical Education and Sport, Faculty of Sports Sciences, Namık Kemal University, Tekirdağ, Turky Turkey; 6 Institute of Sport Sciences, The Jerzy Kukuczka Academy of Physical Education, Katowice, Poland; 7 Research Laboratory, Exercise Physiology and Physiopathology: from Integrated to Molecular “Biology, Medicine and Health” (LR19ES09), Faculty of Medicine of Sousse, University of Sousse, Sousse, Tunisia; 8 Department of Health and Motricity, “Constantin Brâncuşi” University of Târgu-Jiu, Târgu- Jiu, Romania

**Keywords:** physical fitness, conditioning, change of direction, decision-making speed, PACES, RPE, Health care, Neuroscience, Physiology

## Abstract

Agility is a key determinant of soccer performance, combining mechanical change of direction (COD) ability with perceptual and decision-making processes. This study compared the effects of reactive agility (RA) and planned agility (PA) training on physical performance, internal load, and enjoyment in youth soccer players during the in-season period. In a randomized, volume-matched, 8-week intervention, 18 under-16 male players were assigned to a RA (RAG) or PA (PAG) training group. Pre- and post-testing included 10- and 20-m sprints, reactive agility without and with the ball (RAT, RATB), COD and COD with the ball (CODB), countermovement jump (CMJ), balance, and endurance-intensive fitness. Rating of perceived exertion (RPE) and the Physical Activity Enjoyment Scale (PACES) were recorded after each session. Both groups showed significant (*p* < 0.05) pre-post improvements in S10, S20, COD, CODB, CMJ, balance, and endurance-intensive fitness, with no significant group × time interaction for these outcomes. In contrast, large time × group interactions were observed only for RAT and RATB (both *p* < 0.001; ηp^2^ = 0.519 and 0.762, respectively), with RAG being 2.3% and 2.0% faster than PAG, respectively. PACES scores were significantly higher in RAG (*p* < 0.001; d = 9.64), while RPE did not differ between groups (*p* > 0.05). This randomized, volume-matched, in-season comparison demonstrates that stimulus-driven RA provides additional task-specific gains in reactive agility and higher enjoyment, without increasing RPE, compared with PA in youth soccer. Accordingly, integrating brief weekly RA blocks (cue-rich, game-representative drills, with and without the ball) alongside PA may further optimize agility adaptations and adherence in youth soccer.

## Introduction

Soccer performance reflects a complex interplay of physical and cognitive abilities, relying on explosive strength, linear speed, and change of direction (COD) capacity^1,2^, as well as motor control, perception, and cognitive functioning^3^. Although only 15–20% of high-intensity efforts involve repositioning, most actions are tactical and technical and are dictated by game context^4,5^. Elite matches typically include ~ 305 ± 50 CODs^6^, requiring rapid adaptation to external stimuli such as ball trajectory, teammate runs, and opponent positioning^7^. These demands highlight the importance of agility, which integrates both physical and perceptual-cognitive components.

Traditionally, agility was defined as the ability to change direction rapidly, emphasizing speed, coordination, and strength^8^. It is now recognized as distinct from pre-planned COD speed (i.e., planned agility; PA)^9^. PA relies mainly on physical factors such as linear sprinting, strength, and COD technique^10^, but does not capture the unpredictability of match situations^4^. Reactive agility (RA), by contrast, requires perception and decision-making in response to unpredictable stimuli, making it particularly relevant in invasion sports where anticipation, reaction speed, and rapid action selection are critical^9,11,12^.

PA and RA interventions appear to produce complementary adaptations. PA programs (e.g., structured COD drills or multidirectional sprinting) improve timed COD tasks, sprint speed, and jump/strength performance in youth soccer, but transfer to perceptual-cognitive skills and on-ball performance under uncertainty is limited^13–16^. RA training (e.g., COD drills with unpredictable visual or game stimuli) yields larger improvements in reactive-agility outcomes and may transfer to technical performance such as dribbling and ball control^16,17^. Evidence from other sports (e.g., youth volleyball) also supports the cross-sport applicability of RA interventions for visuomotor and reactive-agility improvements^18^. Accordingly, reviews suggest that reactive tasks may offer greater ecological validity for perceptual-cognitive development, whereas PA remains important for building the strength/power and mechanical foundations underpinning rapid direction changes^7,19^. Consistent with this perspective, recent approaches in soccer training increasingly emphasize stimulus-based tasks that better replicate the perceptual and decision-making demands of competition. In this context, RA has been shown to better discriminate skill levels in adolescent soccer players than PA tests, supporting its competitive relevance^20^. Reviews further indicate that RA integrates perceptual and neurological demands largely absent from pre-planned tasks^21^.

Despite these promising findings, most studies have examined PA and RA independently or focused primarily on measurement reliability, rather than directly comparing their training effects on physical performance and psychosocial responses in youth soccer^7,22^. Moreover, although psychosocial factors such as enjoyment and perceived exertion (RPE) may influence engagement and adherence^23^, their responses to RA versus PA training remain underexplored. Therefore, the present study aimed to compare the effects of 8 weeks of stimulus-driven RA training (using external visual cues) versus PA training on linear sprinting, COD (with and without the ball), reactive agility (with and without a ball), vertical jump, balance, endurance-intensive fitness, RPE, and enjoyment in U16 soccer players. We hypothesized that both groups would improve general performance when dose-matched, but that the RA group would show greater improvements in reactive agility (with and without the ball) and higher enjoyment, without higher RPE, compared with the PA group.

## Materials and methods

### Participants

**Fig. 1:**
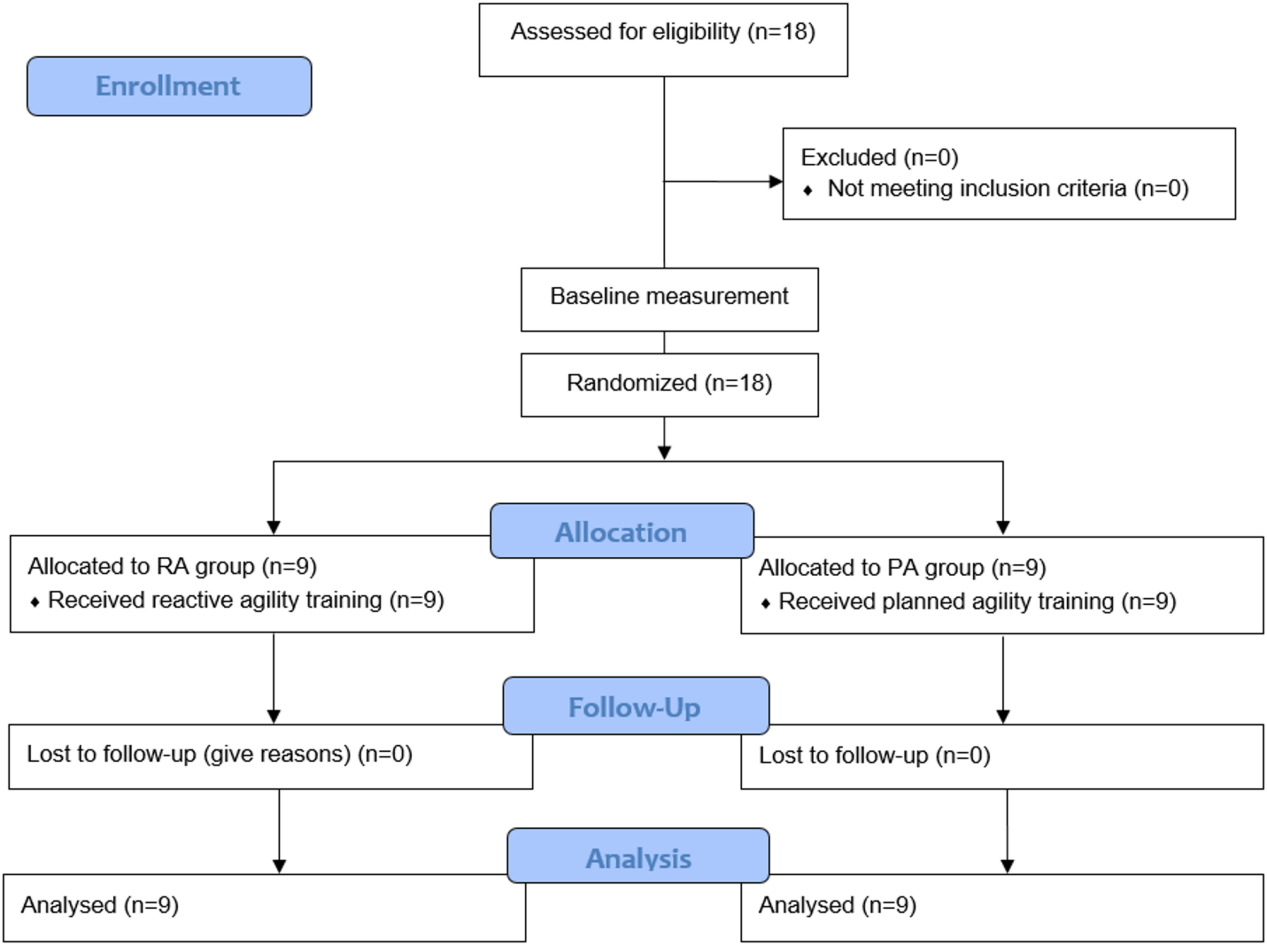
CONSORT (Consolidated standards of reporting trials) Flow diagram of the progress through the phases of a randomized trial of two groups.

Before recruitment, an a priori sample-size estimation was conducted using G*Power (version 3.1.9.7; University of Kiel, Germany) for a repeated-measures ANOVA (within-between interaction; group × time)^24^. With α = 0.05, power (1−β) = 0.80, and an expected large interaction effect (f = 0.40), the minimum required sample was 16 participants (8 per group). To allow for potential attrition, eighteen young male soccer players volunteered to participate in the study. Random allocation was conducted using randomly permuted blocks matched by baseline reactive agility time without the ball, which resulted in the assignment of participants to either an RA group using BlazePod™ (RAG; *n* = 9) or a PA group (PAG; *n* = 9). Randomization was generated using a macro developed in Microsoft Excel (Microsoft Corp., Redmond, WA, USA) by an investigator not involved in testing. The allocation list was stored in a password-protected file and disclosed only after baseline assessment. Participants were identified using coded IDs during all testing sessions. Inclusion criteria were: (a) a minimum of five years of soccer experience and regular participation in club training routines; (b) absence of severe musculoskeletal injuries in the past year; and (c) absence of mild-to-moderate injuries in the past month. All participants were screened and confirmed to be injury-free prior to preliminary testing. To be included in the final analysis, participants were required to complete at least 90% of all training and testing sessions. Figure 1 presents the CONSORT flow diagram of the study.

All participants regularly competed at Algerian U16 regional-level championships, adhering to a weekly microcycle of four training sessions (~ 60–90 min each), with one official match held every Sunday. The participants’ characteristics are presented in Table 1. Written consent was obtained from the participants and their parents after they were thoroughly informed about the study purpose and potential risks. The research protocol was approved by the local ethics committee (approval number: 011/2023; approval date: September 20, 2023) and conducted in accordance with the Declaration of Helsinki.

**Table 1:** Participants’ demographic and anthropometric characteristics.

RAG (*n* = 9)	Age (years)	PHV (years)	APHV (years)	Height (cm)	Body mass (kg)	LLL (cm)	BMI (kg m^−2^)
	14.2 ± 0.4	-0.7 ± 0.7	14.9 ± 0.5	163.0 ± 10.0	49.4 ± 6.7	84.4 ± 5.4	18.5 ± 1.2
PAG (*n* = 9)	14.4 ± 0.5	-0.9 ± 0.6	15.3 ± 0.4	157.7 ± 8.8	49.5 ± 8.7	81.7 ± 4.9	19.9 ± 3.6

The values are expressed as mean and SD.

*BMI* body mass index, *RAG* reactive agility group, *PAG* planned agility group, *PHV* peak height velocity, *APHV*: age at peak height velocity.

### Experimental design

This study adopted a repeated-measures design with randomized allocation to the training intervention. Subjects were assigned to either the RAG or the PAG. Both protocols were matched for weekly and total training volumes, in addition to their regular soccer training regimen. Given the players’ competitive level, the timing within the season, and the need for youth athletes to optimize performance for upcoming matches, the authors assigned participants to two training groups (RAG and PAG) and did not include a control group. Implementing a control condition was deemed unethical in this context, as withholding structured training could impair players’ performance and jeopardize club success across the fixture schedule. This rationale aligns with practices in clinical research when an established, efficacious treatment is available^25^. The study was conducted during the in-season soccer period (from February to April, 2024). Overall, it lasted 10 weeks and consisted of 1 week of pre-testing, 8 weeks of specific training, and 1 week of post-testing.

Participants’ physical fitness was evaluated through a test battery that included 10 m and 20 m linear sprints (S10 and S20), the 15 m COD tests without and with the ball (COD-15 and CODB-15, respectively), the reactive agility tests without and with the ball (RAT and RATB, respectively), the countermovement jump (CMJ), the Y balance test, and the multistage 20-meter shuttle run test. Outcome assessments were performed by the same team of assessors, who were blinded to group allocation. Testing sessions took place approximately 48 h after matches or high-load training sessions to control for residual fatigue and were organized over three evenings (i.e., Tuesday, Wednesday, and Thursday) between 18:00 and 20:00 h on a synthetic turf pitch under consistent environmental conditions (temperature: 17–22 °C, humidity: ~67 ± 2%). Acceleration/speed, COD-15, CODB-15, and jump tests were conducted on the first evening; RAT, RATB, and balance tests were completed on the second; and the endurance-intensive fitness test was performed on the third. Standardized 15-minute warm-ups preceded each session and included jogging, mobility drills, and dynamic movements (e.g., 2 sets of 2 short sprints and zigzag runs of 10 and 15 m). With the exception of the shuttle run, which was executed only once, all tests were performed twice, with the highest value retained for analysis. A two-minute rest separated attempts, while five minutes of rest were given between different tests. Data analysis was performed using anonymized group labels (i.e., Group A/B) to maintain analyst blinding.

To enhance familiarity and ensure correct execution, all participants underwent two orientation sessions held 48 h apart during the week preceding the training intervention. These sessions covered test procedures, training formats, and the use of subjective rating tools such as the Borg Rating of Perceived Exertion scale^26^ and the Physical Activity Enjoyment Scale (PACES)^27^. The reliability of the physical tests (i.e., intraclass correlation coefficient (ICC)), coefficient of variation [CV]) was calculated using the results from the familiarization sessions and those obtained during the pre-test. Anthropometric measurements were also taken during this period: body mass (± 0.1 kg) was measured using a calibrated digital scale (OHAUS, Florham Park, NJ, USA), and height and lower leg length (LLL) were recorded using a stadiometer (± 0.01 m); body mass index (BMI) was subsequently calculated. Maturity status was determined using the anthropometric prediction equation of Mirwald et al.^28^, which estimates maturity offset expressed as the number of years from peak height velocity (PHV), based on leg length, sitting height, chronological age, stature, and body mass.

The agility training interventions were incorporated alongside the athletes’ regular weekly routines. Adherence was closely monitored by the research team. Participants were instructed to fill out a session training log immediately following each intervention, documenting perceived exertion and enjoyment levels via the RPE and PACES tools. All training and evaluation sessions were overseen by the same team to preserve procedural uniformity. To limit external influences, participants were asked to avoid vigorous activity the day before testing and to maintain consistent sleep and training patterns throughout the study.

### Procedures

#### Linear sprinting assessment

To assess acceleration/speed, participants performed the 10 and 20 m linear sprint tests at maximal effort. Sprint time (seconds) was recorded using a series of paired photocells (Globus, Microgate, Bolzano, Italy). The photocells were placed 0.2 m above the ground at the start line, with the front foot positioned 0.5 m behind the first gate, and 1.0 m above the ground at the 10-m and 20-m marks^29^. The ICCs for S10 and S20 were 0.968 and 0.968, respectively, and their corresponding CVs were 2.06% and 1.08%.

#### Change of direction assessment

The COD ability was assessed using a 15-m COD run performed both without and with a ball, following Mujika et al.^30^. For the no-ball condition, players started 3 m behind the first timing gate (Globus, Microgate, Bolzano, Italy), accelerated 3 m, negotiated a 3 m slalom delineated by three 1.6-m poles spaced 1.5 m apart, cleared a 0.5-m hurdle located 2 m beyond the final pole, and then sprinted the remaining 7 m to the finish timing gate. The ball condition replicated this layout but required participants to play the ball under the hurdle while jumping over it, then direct the ball toward one of two small goals positioned diagonally 7 m to the left or right of the hurdle before completing the sprint to the finish. The ICCs for COD-15 and CODB-15 were 0.982 and 0.990, with corresponding CVs of 1.45% and 0.74%.

#### Reactive agility assessment

**Fig. 2:**
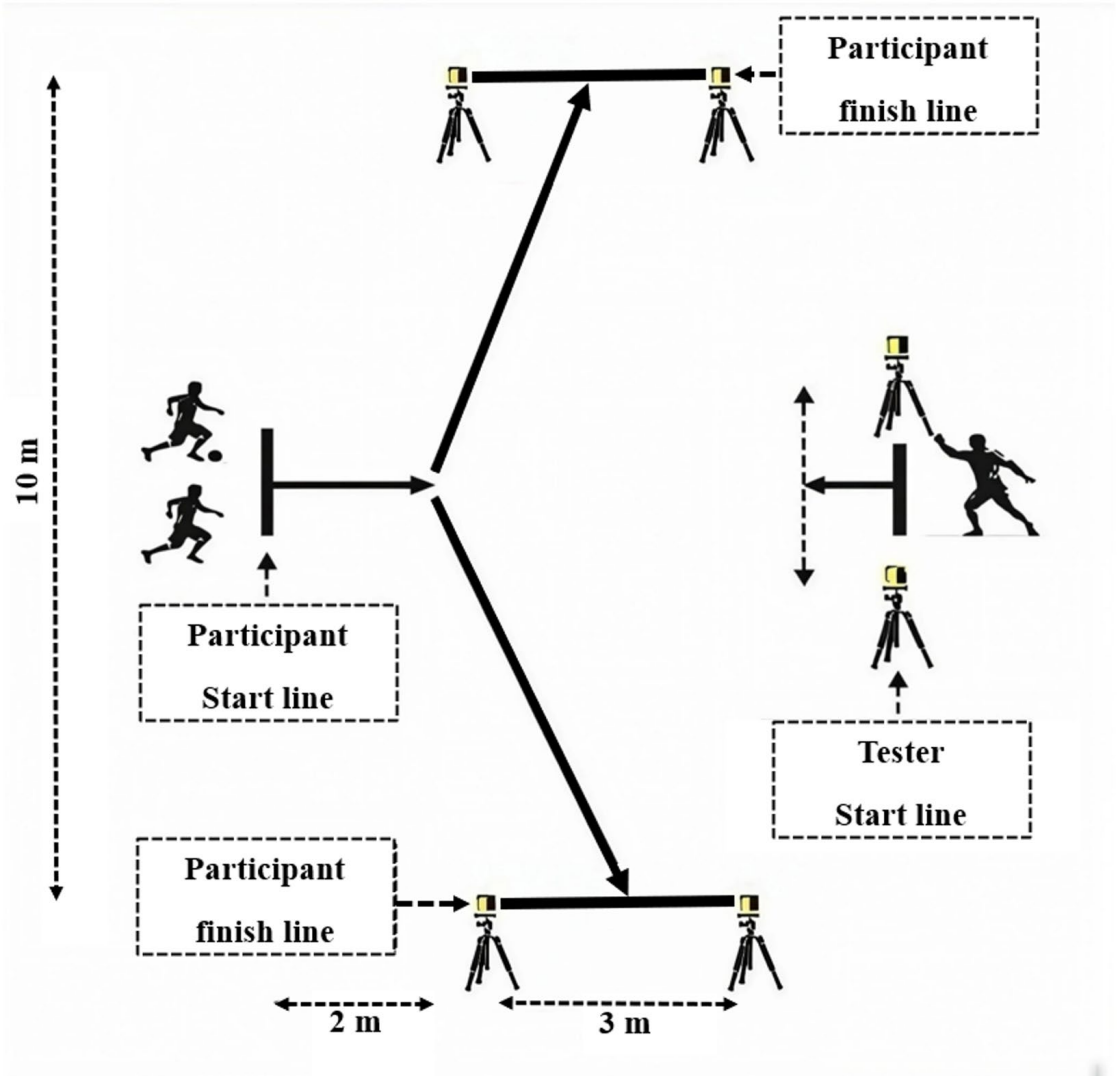
Schematic representation of the reactive agility test without (RAT) and with a ball (RATB).

Reactive agility was assessed using two formats: the RAT was performed without a ball, whereas the RATB required participants to perform the same task while controlling a ball^16,31^ (Fig. 2). In both tests, the participant stood behind a start line, while a tester (acting as an opponent) was positioned just behind the start-line timing gate (Globus, Microgate, Bolzano, Italy), directly facing the participant. Two finish timing gates (photocells positioned 0.4 m above the ground) were placed 2 m in front of the start line and 5 m to the left and right, resulting in a 10 m distance between the two finish gates. Each trial was initiated by the tester stepping forward approximately 0.5 m, which served as the “go” signal. The tester then provided one of four standardized lower-limb cues to indicate the required change of direction: (1) a single right-foot step signaled a cut to the left; (2) a single left-foot step signaled a cut to the right; (3) a rapid right-left step sequence signaled a cut to the right; and (4) a rapid left-right step sequence signaled a cut to the left. The participant was required to read the cue in real time, accelerate, change direction accordingly, and sprint through the correct finish gate to stop the timer. Each participant completed all four cue options in randomized order and repeated the set twice (two series) for a total of eight trials. Performance was expressed as the mean time across all valid trials for each condition. For RAT and RATB, the ICCs were 0.897 and 0.791. The CVs for the same conditions were 1.72% and 2.13%.

#### Jump assessment

The CMJ was conducted following the method described by Bosco et al.^32^ and measured using an infrared jump system (Optojump; Microgate, Bolzano, Italy). Participants started in a normal bipedal stance, performed a rapid countermovement to approximately 90° of knee flexion, and immediately jumped vertically as high as possible. To eliminate the influence of arm swing, participants were instructed to place their hands on their hips throughout the test. Knees and ankles were required to be fully extended on takeoff and during tiptoe landing. The ICC and CV for the CMJ were 0.841 and 4.28%, respectively.

#### Dynamic balance assessment

Dynamic balance was assessed using the Y-balance test, following the procedure described by Plisky et al.^33^. For each limb and each reach direction, the maximal reach distance (cm) was measured and used for analysis. A composite score (%) was then calculated for each leg and retained for further analysis^33^. The ICC and CV for the test were 0.932 and 0.30%, respectively.

#### Endurance-intensive fitness assessment

The multistage 20-meter shuttle run test^34^ is a progressive, maximal aerobic field test used to estimate cardiorespiratory fitness. Participants ran back and forth between two lines set 20 m apart, synchronized with audio signals that increase in frequency each minute. The test started at a speed of 8 km·h^−1^ and increased by 0.5 km·h^−1^ at each stage until the participant could no longer maintain the pace. The final speed reached (i.e., maximal aerobic velocity [MAV]) was used to estimate maximum oxygen uptake (VO2max) using the following Eq. 3^4^: VO2max (ml·kg^−1^·min^−1^) = 31.025 + (3.238 × MAV) - (3.248 × Age) + (0.1536 × MAV × Age). This test is widely recognized for its validity, reliability, and practicality in both youth and adult populations^34^.

#### Rating of perceived exertion

Following each agility-training session, participants rated their exertion level using Borg’s 10-point Likert scale (0–10 arbitrary units [AU]) with 0 corresponding to “rest” effort and 10 to “maximal” effort^26^.

#### Enjoyment assessment

The original 18-item PACES is a self-report questionnaire developed to assess an individual’s enjoyment of physical activity^27^. Each item is rated on a 7-point bipolar Likert scale, with statements reflecting positive and negative feelings toward the activity (e.g., “I enjoy it” vs. “I hate it”). Scores range from 18 to 126, with higher scores indicating greater enjoyment. The PACES questionnaire was completed immediately following the specific training, using a paper-and-pencil format.

The items of the PACES questionnaire were translated into the native language of the participants by a panel of three professional English-Native language translators with proven expertise in psychological scale translation. Each translator independently translated all items, and the consistency across versions was evaluated. For each item, agreement among the translators was scored as 1 when all versions matched, and as 0 in the case of any discrepancies. Overall agreement across all items was then analyzed, yielding an inter-translator consistency coefficient of 0.94. Post-translation internal consistency of the PACES was good (Cronbach’s α = 0.91; 18 items; *n* = 18), computed after reverse-scoring negatively worded items.

### Training programs

The 8-week agility training intervention was conducted twice per week (Wednesday and Friday), immediately after the standardized warm-up at the beginning of regular team sessions. Both programs were matched for volume, intensity, and exercise structure but differed in task nature: the PAG performed predetermined movement patterns, whereas the RAG responded to visual cues generated by BlazePod™ devices (Play Coyotta Ltd., New York, USA). Each session consisted of four multidirectional, high-intensity drills (i.e., 5–5 COD [10 m], 4-cone box [16 m], half-T drill [20 m], and star drill [30 m]) designed to enhance acceleration, deceleration, and COD ability. All exercises were performed at maximal intensity (all-out) to ensure high neuromuscular engagement^35^ and were supervised by a certified strength and conditioning specialist to guarantee correct technique and maximal effort. Players completed 2–3 sets of 2–6 repetitions per exercise, with 25–60 s of passive recovery between repetitions and 3–4 min between sets, maintaining a work-to-rest ratio of approximately 1:10 − 1:12 to preserve movement quality and avoid pacing^36^. The estimated total distance covered per session ranged from 270 to 540 m, depending on drill type and week progression. RPE was collected after each specific agility training session to monitor internal load, and full exercise details are presented in Table 2. No participants withdrew during the intervention period, and no dropouts occurred due to injury.

**Table 2:** Descriptive characteristics of the training program performed by both experimental groups.

Weeks	5-0-5 COD (10 m)			4-Cone Box (16 m)			Half-T Drill (20 m)			Star Drill (30 m)			Estimated distance (m)/ session	RPE / Week	
	Sets×Reps	*r* (s)	*R* (min)	Sets×Reps	*r* (s)	*R* (min)	Sets×Reps	*r* (s)	*R* (min)	Sets×Reps	*r* (s)	*R* (min)		RAG	PAG
Week 1	2 × 4	25–30	3	2 × 4	35–40	3	2 × 3	35–40	3				~ 320	5.06	5.17
Week 2	2 × 5	25–30	3	2 × 6	35–40	4				2 × 2	60	4	~ 400	5.62	5.84
Week 3	3 × 4	35–40	4	3 × 4	35–40	4	2 × 4	35–40	4				~ 460	6.17	6.45
Week 4	3 × 4	35–40	4				3 × 3	35–40	4	2 × 3	60	4	~ 480	6.78	7.17
Week 5	2 × 4	25–30	3				2 × 3	35–40	3	2 × 2	60	4	~ 320	5.28	5.39
Week 6				3 × 4	35–40	4	2 × 4	35–40	4	2 × 3	60	4	~ 475	6.62	6.95
Week 7				3 × 4	35–40	4	3 × 4	35–40	4	2 × 2	60	4	~ 540	7.33	7.56
Week 8	2 × 3	25–30	3	2 × 3	35–40	3	2 × 3	35–40	3				~ 270	4.39	4.33

Reps: repetitions; r: rest between repetitions; R: rest between sets; RPE: rate of perceived exertion; RAG: reactive agility group; PAG: planned agility group.

### Statistical analysis

All data are expressed as mean ± standard deviation (SD). To assess whether the variables followed a normal distribution, the Shapiro-Wilk test was performed, while Levene’s test was used to evaluate homogeneity of variance. The within-subject reliability (individual variability across training sessions) and between-session reliability (group variability across training sessions) of RPE and PACES scores were calculated for each group and expressed as CV% with 95% confidence interval (CI), using the spreadsheet developed by Hopkins^37^. An independent samples t-test was conducted to assess differences in internal load responses and enjoyment scores between the two groups. Α 2 × 2 (time × group) mixed ANOVA with repeated measures was used to examine the main and interaction effects across all variables. For post hoc analyses, the Bonferroni or Games-Howell tests were applied depending on variance homogeneity. To support clarity and align analyses with the study aims, outcomes were interpreted using a pre-specified hierarchy. Reactive agility outcomes were treated as primary outcomes, whereas COD/CODB, linear sprint, CMJ, balance, endurance-intensive fitness, and psychosocial/internal load measures (PACES and session RPE) were treated as secondary outcomes. Effect sizes for ANOVA were reported as partial eta-squared (ηp^2^) and interpreted as follows: small (0.01 < ηp^2^ < 0.06), medium (0.06 ≤ ηp2<0.14), and large (ηp^2^ ≥ 0.14)^38^. Cohen’s d was calculated to quantify the magnitude of between-group differences and was classified as follows: trivial (< 0.20), small (0.20 ≤ d < 0.50), medium (0.50 ≤ d < 0.80), and large (≥ 0.80)^38^. In addition to p-values and effect sizes, percentage changes (Δ%) were reported to facilitate practical interpretation of the magnitude of change. Given the number of outcomes assessed, interpretation was guided primarily by the outcome hierarchy and the consistency of group × time interaction effects rather than isolated statistically significant findings among secondary outcomes. Statistical analyses were conducted using SPSS version 26 for Windows (IBM Corp, Armonk, NY, USA), with the significance level set at *p* ≤ 0.05.

## Results

PAG showed no significant differences from RAG at baseline in chronological age, PHV, APHV, height, LLL, body mass, or BMI (all *p* > 0.05; Table 1). RPE during the training sessions did not differ between groups (*p* > 0.05). Within-subject and between-session variability in PACES scores was significantly lower during RAG than during PAG (Table 3). Across sessions, enjoyment scores were significantly higher in RAG than PAG (*p* < 0.05; Table 3).

**Table 3:** Internal load responses and enjoyment scores of the reactive (*n* = 9) and planned (*n* = 9) agility training groups.

Variable	Group	Mean	SD	95% CI	Variability (CV%)		Group comparaison		
					Intersession, 95% CI	Intrasubject, 95% CI	t	p	d
RPE (AU)	RAG	5.9	0.5	5.5–6.3	11.3–15.0	16.3–21.3	0.937	0.363	0.44
	PAG	6.1	0.4	5.8–6.4	10.1–14.0	18.0-21.2			
PACES (AU)	RAG	89.7	2.3	87.9–91.5	3.2–4.2	2.3–3.2	20.44†	< 0.001	9.64
	PAG	64.1	3.0	61.9–66.4	5.1–6.5	5.6–7.8			

The values are expressed as mean and SD with 95% confidence interval (95% CI) of the 16 training sessions performed during the intervention period by both agility training groups. RPE: rate of perceived exertion; AU: arbitrary unit; PACES: physical activity enjoyment scale scores d: cohen’s d. The intersession CV% represents the mean variability of the training load responses with the group across the intervention period and expressed as coefficient of variation; intrasubject CV% represents the mean variability of the training load responses and enjoyment scores of the individual subjects across the intervention period and expressed as coefficient of variation.

†A significant intergroup difference. The statistical significance level was set at *p* ≤ 0.05.

There were significant main effects of time for all measured variables (all *p* < 0.05; Tables 4 and 5). Large time × group interactions were observed only for RAT and RATB (both *p* < 0.001), with RAG being 2.3% and 2.0% faster than PAG, respectively. No significant main effects of group were found for any variable (all *p* > 0.05; Tables 4 and 5).

**Table 4:** Changes in linear sprint, change of direction with and without a ball and reactive agility sprint with and without a ball performances in both groups (*n* = 18).

Variable	Group	Pre	Post	d (*p*)	∆ (%)	ANOVA
S10 (s)	RAG	1.93 ± 0.15	1.84 ± 0.18†	0.543 (< 0.001)	-4.4 ± 2.9	Time: F_1,18_=62.915; *p* < 0.001; ηp^2^ = 0.777 Group: F_1,18_=0.108; *p* = 0.746; ηp^2^ = 0.006 Interaction: F_1,18_=0.256; *p* = 0.619; ηp^2^ = 0.014
	PAG	1.95 ± 0.15	1.87 ± 0.15†	0.533 (< 0.001)	-3.8 ± 1.8	
S20 (s)	RAG	3.69 ± 0.16	3.58 ± 0.17†	0.681 (< 0.001)	-2.8 ± 1.6	Time: F_1,18_=47.612; *p* < 0.001; ηp^2^ = 0.726 Group: F_1,18_=0.050; *p* = 0.825; ηp^2^ = 0.003 Interaction: F_1,18_=0.123; *p* = 0.730; ηp^2^ = 0.008
	PAG	3.66 ± 0.19	3.57 ± 0.17†	0.499 (0.001)	-2.5 ± 1.8	
COD-15 (s)	RAG	4.11 ± 0.42	3.85 ± 0.32†	0.696 (< 0.001)	-6.1 ± 3.1	Time: F_1,18_=82.923; *p* < 0.001; ηp^2^ = 0.822 Group: F_1,18_=0.005; *p* = 0.946; ηp^2^=0 Interaction: F_1,18_=0.062; *p* = 0.806; ηp^2^ = 0.003
	PAG	4.13 ± 0.35	3.86 ± 0.31†	0.817 (< 0.001)	-6.5 ± 2.4	
CODB-15 (s)	RAG	5.41 ± 0.36	5.20 ± 0.40†	0.552 (< 0.001)	-4.0 ± 2.9	Time: F_1,18_=59.922; *p* < 0.001; ηp^2^ = 0.769 Group: F_1,18_=0.004; *p* = 0.947; ηp^2^=0 Interaction: F_1,18_=1.169; *p* = 0.294; η^2^*p* = 0.061
	PAG	5.44 ± 0.35	5.15 ± 0.33†	0.853 (< 0.001)	-5.2 ± 2.5	
RAT (s)	RAG	2.32 ± 0.11	2.25 ± 0.09†	0.697 (< 0.001)	-3.1 ± 1.5	Time: F_1,18_=56.769; *p* < 0.001; ηp^2^ = 0.759 Group: F_1,18_=0.516; *p* = 0.482; ηp^2^ = 0.028 Interaction: F_1,18_=19.452; *p* < 0.001; ηp^2^ = 0.519
	PAG	2.32 ± 0.07	2.30 ± 0.06†	0.307 (0.040)	-0.8 ± 0.3	
RATB (s)	RAG	2.82 ± 0.08	2.73 ± 0.07†	1.197 (< 0.001)	-3.2 ± 0.7	Time: F_1,18_=267.526; *p* < 0.001; ηp^2^ = 0.937 Group: F_1,18_=0.242; *p* = 0.815; ηp^2^ = 0.013 Interaction: F_1,18_=57.668; *p* < 0.001; ηp^2^ = 0.762
	PAG	2.81 ± 0.09	2.78 ± 0.09†	0.333 (< 0.001)	-1.2 ± 0.3	

The values are expressed as mean and SD.

S10: 10 m sprint; S20: 20 m sprint; COD-15: 15 m change of direction sprint without a ball; CODB: 15 m change of direction sprint with a ball; RAT: reactive agility sprint without a ball; RATB: reactive agility sprint with a ball; RAG: reactive agility group; PAG: planned agility group; d: cohen’s d; Δ: percentage changes; ηp^2^: partial eta squared. †A significant within group difference. The statistical significance level was set at *p* ≤ 0.05.

**Table 5:** Changes in jumping, balance and aerobic power performances in both groups (*n* = 18).

Variable	Group	Pre	Post	d (*p*)	∆ (%)	ANOVA
CMJ (cm)	RAG	31.4 ± 3.2	35.1 ± 2.2†	1.334 (< 0.001)	12.3 ± 9.3	Time: F_1,18_=50.876; *p* < 0.001; ηp^2^ = 0.739 Group: F_1,18_=2.416; *p* = 0.137; ηp^2^ = 0.118 Interaction: F_1,18_=0.212; *p* = 0.651; ηp^2^ = 0.012
	PAG	30.1 ± 2.4	33.3 ± 2.0†	1.450 (< 0.001)	11.0 ± 6.6	
Balance	RAG	98.3 ± 1.2	100.5 ± 1.8†	1.458 (< 0.001)	2.2 ± 0.8	Time: F_1,18_=197.147; *p* < 0.0001; ηp^2^ = 0.916 Group: F_1,18_=2.289; *p* = 0.148; ηp^2^ = 0.113 Interaction: F_1,18_=0.158; *p* = 0.696; ηp^2^ = 0.009
	PAG	99.1 ± 0.6	101.2 ± 1.0†	2.547 (< 0.001)	2.1 ± 0.6	
MAV (km.h^−1^)	RAG	11.7 ± 0.6	12.3 ± 0.7†	1.032 (0.009)	5.7 ± 3.8	Time: F_1,18_=16.754; *p* = 0.001; ηp^2^ = 0.482 Group: F_1,18_=0.273; *p* = 0.608; ηp^2^ = 0.015 Interaction: F_1,18_=0.138; *p* = 0.714; η^2^*p* = 0.008
	PAG	11.9 ± 1.1	12.4 ± 0.7†	0.597 (0.025)	5.1 ± 7.7	
VO2max (ml.min^− 1^.kg^− 1^)	RAG	48.1 ± 3.2	51.7 ± 3.8†	1.033 (0.010)	7.6 ± 5.1	Time: F_1,18_=16.595; *p* = 0.001; ηp^2^ = 0.479 Group: F_1,18_=0.091; *p* = 0.766; ηp^2^ = 0.005 Interaction: F_1,18_=0.114; *p* = 0.740; ηp^2^ = 0.006
	PAG	48.9 ± 6.6	52.0 ± 4.2†	0.560 (0.024)	7.3 ± 10.7	

The values are expressed as mean and SD.

CMJ: countermovement jump; MAV: maximal aerobic velocity; VO2max: maximal oxygen consumption; RAG: reactive agility group; PAG: planned agility group; d: cohen’s d; Δ: percentage changes; ηp^2^: partial eta squared. ^†^A significant within group difference. The statistical significance level was set at *p* ≤ 0.05.

## Discussion

This study examined the effects of RA training and PA training on multiple performance, internal load, and physical enjoyment outcomes in youth soccer players during the in-season period. Both training modalities produced significant improvements across all tested variables, yet the key distinction emerged in reactive agility, where RAG elicited superior enhancements in both RAT and RATB. Furthermore, RAG resulted in higher enjoyment while maintaining similar RPE compared to PAG.

The superior gains in RAT and RATB following RA training highlight the importance of integrating stimulus-driven, decision-dependent demands into agility development. Agility performance depends not only on the mechanical ability to change direction but also on perceptual and decision-making processes^7–9^. In the present study, the RA program incorporated unpredictable visual cues and real-time action selection, which likely increased the specificity of practice relative to the reactive agility tests (i.e., RAT and RATB). Accordingly, the observed improvements should be interpreted primarily as task-specific adaptations to training under stimulus-response conditions rather than as direct evidence of enhanced perceptual-cognitive processing or neural adaptation. Although perceptual–cognitive mechanisms are plausible, they remain inferred here because no direct measures of perceptual skill, decision-making speed, or cognitive function were collected. Practically, the between-group differential change in RAT and RATB (supported by group × time interactions) indicates that RA training produced additional task-specific improvements beyond those observed with PA under matched dose, consistent with the principle of training specificity. Importantly, the presence of the ball did not attenuate the benefits of RA training. Together, these findings support the practical value of stimulus-driven training when the desired outcome is reactive agility performance, with or without the ball.

The greater PACES scores observed during RA training, despite equivalent RPE to PAG, are consistent with evidence suggesting that gamified and uncertainty-rich environments improve intrinsic motivation and engagement^39,40^. The higher enjoyment observed in RAG may reflect the more interactive, variable, and decision-oriented nature of the drills. However, qualitative differences between RA and PA formats (greater task variability and unpredictability in RA vs. more pre-planned structure in PA) may influence engagement independent of training “superiority.” Therefore, enjoyment differences may be partly attributable to novelty/variety (“gamification”) effects and contextual factors. This study did not assess psychological mediators (e.g., autonomy, competence, relatedness), so such explanations should be considered interpretive rather than demonstrated. Accordingly, enjoyment results should be interpreted as reflecting training experience and tolerability, and future work should control for novelty/variability and assess psychological mediators to clarify why enjoyment differs.

In contrast, the non-significant group differences for S10, S20, COD-15, CODB-15, CMJ, balance, and endurance-intensive fitness likely reflect the shared neuromuscular content of both interventions. Both RA and PA training involved repeated acceleration-deceleration patterns and multidirectional movement mechanics under matched volume and intensity^41,42^. When external load and movement exposure are similar, comparable improvements in physical parameters are expected^7,43^. Furthermore, the absence of additional gains from RA training in purely physical metrics supports the specificity principle. RA training may confer its greatest advantage when the outcome requires rapid stimulus-response coupling (i.e., reactive agility), but it does not necessarily improve linear speed or muscular power^16,43^. Therefore, outcomes showing primarily time effects (with no clear between-group differential change) should be interpreted more conservatively and not overemphasized relative to the primary reactive agility outcomes. The comparable RPE between groups further suggests that both programs imposed similar physiological loads, yet RAG’s higher enjoyment highlights its potential motivational and engagement value, which may be important for sustaining performance throughout the competitive season. Taken together, these findings suggest that dose-matched PA and RA can improve general physical capacities in-season, while RA provides added benefit for reactive-agility performance and perceived enjoyment.

### Practical implications

Integrating planned and reactive agility trainings within the weekly microcycle appears most appropriate for in-season youth soccer. PA training sustains the foundational mechanical capacities that underpin COD (strength-speed-technique), whereas RA training preferentially develops reactive, stimulus-driven agility performance and engagement. Practically, we recommend one to two brief weekly RA blocks, using cue-rich, game-representative tasks (e.g., light- or coach/opponent-cued direction changes, reactive dribbling duels, or small-sided games with limited planning time), implemented with and without the ball. This approach may enhance match-relevant reactive agility and enjoyment without increasing perceived exertion, while PA maintains the physical base; periodic RAT/RATB assessments may be used to monitor adaptation.

### Limitations

Several limitations should be acknowledged when interpreting these findings. First, the study sample was relatively small and composed exclusively of amateur U16 male soccer players from a single club, which limits generalizability to other age groups, sexes, or competitive levels. Although the a priori power analysis supported the chosen sample size for detecting large group × time interaction effects, the inclusion of only 18 male players from one competitive level and cultural context limits external validity and may reduce sensitivity to smaller effects. Second, the absence of a non-training control group limits causal attribution, particularly given the large main effects of time across outcomes. Thus, pre-post improvements may reflect both intervention effects and general in-season influences (routine training, match exposure, seasonal adaptation), and maturation-related changes in this youth sample (e.g., proximity to PHV) may also have contributed. Therefore, variables showing mainly time effects without a group × time interaction should be interpreted cautiously, whereas training specificity is best supported by outcomes with clear group × time interactions (notably RAT and RATB). Third, the intervention lasted only eight weeks during the in-season period; longer-term or off-season studies may reveal different adaptation magnitudes or retention patterns. Fourth, although RA and PA drills were matched for total volume and intensity, subtle differences in external or internal load could have influenced outcomes. Fifth, enjoyment was assessed using a translated version of the PACES questionnaire, and while the translation was validated by expert consensus, cultural nuances in interpreting enjoyment may remain. Given that enjoyment can be influenced by contextual factors (e.g., coaching style, team culture, group dynamics, and novelty/greater task vaiability), psychosocial findings may be context-dependent and may not generalize across settings. Moreover, the exceptionally large between-group effect size observed for PACES warrants cautious interpretation, as such values are uncommon in behavioral research and may reflect contextual and/or measurement-related influences (e.g., response tendencies, translation/cultural interpretation of items, repeated session-based administration, or team-specific engagement) rather than a purely training-induced effect. In addition, the higher enjoyment in the RA condition may partly reflect novelty and greater task variability relative to PA drills. Lastly, the use of light-based cue systems, though reliable and ecologically valid, may not fully replicate the complexity of opponent-driven stimuli found in competitive matches. Moreover, we did not include direct measures of perceptual skill, decision-making speed, or cognitive function; therefore, mechanistic explanations (e.g., perceptual-cognitive or neural adaptations) should be considered inferred rather than demonstrated. Future research should replicate these findings in larger, mixed-gender, multi-club samples with longer follow-up, include direct perceptual-cognitive measures, monitor maturity status, and further evaluate PACES psychometrics under repeated in-season administration.

## Conclusion

Both RA and PA protocols produced meaningful in-season improvements in acceleration/speed, COD, jumping, balance, and endurance-intensive fitness in youth soccer players. However, only RA training elicited superior gains in RAT and RATB under matched training dose, supporting the added value of stimulus-response training for task-specific reactive-agility performance. RA training also increased enjoyment without increasing RPE, suggesting potential motivational benefits that may help sustain engagement during the competitive season, although contextual and novelty effects cannot be excluded. Collectively, these findings support integrating brief RA blocks alongside PA within the microcycle to target both mechanical and reactive components of agility. Future studies should replicate these results in larger, mixed-gender, multi-club samples, include longer follow-up and direct perceptual-cognitive measures, and examine transfer to match performance.

## Data Availability

Data supporting the findings of this study are available from the corresponding author upon reasonable request.
